# Garlic (*Allium sativum* L.) Bioactives and Its Role in Alleviating Oral Pathologies

**DOI:** 10.3390/antiox10111847

**Published:** 2021-11-21

**Authors:** Minnu Sasi, Sandeep Kumar, Manoj Kumar, Sandhya Thapa, Uma Prajapati, Yamini Tak, Sushil Changan, Vivek Saurabh, Shweta Kumari, Ashok Kumar, Muzaffar Hasan, Deepak Chandran, Radha, Sneh Punia Bangar, Sangram Dhumal, Marisennayya Senapathy, Anitha Thiyagarajan, Ahmad Alhariri, Abhijit Dey, Surinder Singh, Suraj Prakash, Ravi Pandiselvam, Mohamed Mekhemar

**Affiliations:** 1 Division of Biochemistry, ICAR—Indian Agricultural Research Institute, New Delhi 110012, India; minnusasi1991@gmail.com; 2 Quality and Productivity Improvement Division, ICAR—Indian Institute of Natural Resins and Gums, Ranchi 834010, India; sandeep.kumar8@icar.gov.in; 3 Chemical and Biochemical Processing Division, ICAR—Central Institute for Research on Cotton Technology, Mumbai 400019, India; 4 Department of Horticulture, Institute of Agricultural Sciences, Banaras Hindu University, Varanasi 221005, India; sandhyabhu94@gmail.com; 5 Division of Food Science and Postharvest Technology, ICAR—Indian Agricultural Research Institute, New Delhi 110012, India; uma_11103@iari.res.in (U.P.); vivek_11593@iari.res.in (V.S.); 6 Department of Biochemistry, Agriculture University, Kota 324001, India; yaminitak1992@gmail.com; 7 Division of Crop Physiology, Biochemistry and Post-Harvest Technology, ICAR—Central Potato Research Institute, Shimla 171001, India; sushil.changan@icar.gov.in; 8 Centre for Agricultural Bioinformatics, ICAR—Indian Agricultural Statistics Research Institute (IASRI), Library Avenue, New Delhi 110012, India; shwetamgr1@gmail.com; 9 Post-Harvest Technology and Biochemistry, ICAR—Directorate of Onion and Garlic Research, Pune 410505, India; ashokanuraj@gmail.com; 10 Agro Produce Processing Division, ICAR—Central Institute of Agricultural Engineering, Bhopal 462038, India; muzaffar.hasan@icar.gov.in; 11 Department of Veterinary Sciences and Animal Husbandry, Amrita School of Agricultural Sciences, Amrita Vishwa Vidyapeetham University, Coimbatore 642109, India; c_deepak@cb.amrita.edu; 12 School of Biological and Environmental Sciences, Shoolini University of Biotechnology and Management Sciences, Solan 173229, India; radhuchauhan7002@gmail.com (R.); surajpandiar75@gmail.com (S.P.); 13 Department of Food, Nutrition, & Packaging Sciences, Clemson University, Clemson, SC 29634, USA; snehpunia69@gmail.com; 14 Division of Horticulture, RCSM College of Agriculture, Kolhapur 416004, India; sdhumal@msu.edu; 15 Department of Rural Development and Agricultural Extension, College of Agriculture, Wolaita Sodo University, Wolaita Sodo 138, SNNPR, Ethiopia; drsenapathy@wsu.edu.et; 16 Department of Postharvest Technology, Horticultural College and Research Institute, Periyakulam 625604, India; anitha.anitha303@gmail.com; 17 Faculty of Agriculture, Damascus University, Damascus 30621, Syria; ahmadharere@yahoo.com; 18 Department of Life Sciences, Presidency University, 86/1 College Street, Kolkata 700073, India; abhijit.dbs@presiuniv.ac.in; 19 Dr. S. S. Bhatnagar University Institute of Chemical Engineering and Technology, Panjab University, Chandigarh 160014, India; 20 Division of Physiology, Biochemistry and Post-Harvest Technology, ICAR—Central Plantation Crops Research Institute (CPCRI), Kasaragod 671124, India; anbupandi1989@yahoo.co.in; 21 Clinic for Conservative Dentistry and Periodontology, School of Dental Medicine, Christian-Albrecht’s University, 24105 Kiel, Germany

**Keywords:** phytochemicals, garlic, bioactivities, antioxidant, oral care, oral health, oral cancer, oral pathology

## Abstract

Garlic (*Allium sativa* L.) is a bulbous flowering plant belongs to the family of Amaryllidaceae and is a predominant horticultural crop originating from central Asia. Garlic and its products are chiefly used for culinary and therapeutic purposes in many countries. Bulbs of raw garlic have been investigated for their role in oral health, which are ascribed to a myriad of biologically active compounds such as alliin, allicin, methiin, S-allylcysteine (SAC), diallyl sulfide (DAS), S-ally-mercapto cysteine (SAMC), diallyl disulphide (DADS), diallyl trisulfide (DATS) and methyl allyl disulphide. A systematic review was conducted following the PRISMA statement. Scopus, PubMed, Clinicaltrials.gov, and Science direct databases were searched between 12 April 2021 to 4 September 2021. A total of 148 studies were included and the qualitative synthesis phytochemical profile of GE, biological activities, therapeutic applications of garlic extract (GE) in oral health care system, and its mechanism of action in curing various oral pathologies have been discussed. Furthermore, the safety of incorporation of GE as food supplements is also critically discussed. To conclude, GE could conceivably make a treatment recourse for patients suffering from diverse oral diseases.

## 1. Introduction

Natural products and traditional medicines are of substantial importance. Modern medicine may not be the sole antidote for the ailments prevailing today. Therefore, people positively perceive ‘back to nature’ approaches like phytotherapy as plant products are rich in pharmaceuticals [1,2,3]. *Allium sativum*, which is well-known as garlic, belongs to the family Amaryllidaceae. It has been known to humankind for many ages for its bioceutical properties. Garlic is indigenous to central Asia and has long been an important crop in the Mediterranean region and as seasoning in continents like Africa and Europe. India ranks second in garlic production, where the first is China [4].

*Allium sativum* is conventionally employed to cure infections, common cold, diabetes, and heart diseases. Ancient Egyptians used garlic for both culinary and curative purposes. In Egypt, garlic was fed to the working class involved in heavy labour during the construction of the pyramids and in Greece, during the earliest Olympics, it was provided as “performance enhancers” in competitive athletics, meanwhile, among the Romans, garlic was known to purify the arteries [5,6]. In India, the excellent surviving medical book, Charaka- Samhita, suggested the consumption of garlic to treat heart disease and arthritis 1900 years ago [7]. 

Fresh raw garlic bulbs comprise of ∼66% water, ∼27% carbohydrate, ∼2.5% protein, ∼1.3% amino acids, ∼1.6% fiber, fatty acids, phenols, trace minerals and more than 34 (∼2.4%) sulfur-containing compounds [8]. The phytochemicals majorly belong to polyphenols, amino acids, benzenoids, sulfur-containing compounds, fatty acyls, glycerophospholipids, heteroaromatic compounds, indoles, phenol lipids, pyrrolizines, quinolines, steroid derivatives, tetrahydrofurans and other compounds [9]. The beneficial effect of GE on health could be ascribed to the phytochemicals generated during the extraction process, like alliin, methiin and SAC. When garlic is ground, the structure of the sulphur components are altered into various organosulfur compounds. The primary sulfur-containing constituents in whole, intact garlic are the SAC, SAMC, N-alfa-fructosyl arginine, glutamyl 7 cysteines and S allyl cysteine sulfoxides, including alliin [10]. Enzymes in garlic like allinase will convert alliin into allicin, which has antimicrobial action against oral pathogens and confines promise to cure periodontal diseases, dental caries and oral cancers. Volatile compounds in finely ground garlic and essential oil include DAS, DADS, DATS, methyl allyl disulfide, methyl allyl trisulfide, 2-vinyl-1, 3-dithiin, 3-vinyl-1, 2-dithiin [11] and ajoene [12].

Clinically, garlic has been established for reducing blood pressure, cholesterol, and amounts of glucose, as well as for the prohibition of arteriosclerosis and cancer. Lack of regular oral sanitation results in accretion of dental plaque and calculus, which are the critical etiology reasons for gingivitis and periodontitis. Caries is one of the most common oral infectious diseases widespread globally in every segment of population and the development is influenced by dietary components which interfere with etiological agents *Streptococcus sobrinus* and *Streptococcus mutans* [13,14]. In recent years, active research to find generally recognized as safe compounds is ongoing with goal to prevent and to reduce caries and number of foods e.g., in vivo studies of garlic extract has shown potential anticaries activities against pathogen mutans group Streptococci [15,16,17,18,19,20]. Globally, second most common diseases are periodontitis, prevalent at 30 to 50% of United States population. Novel ideas are emerging with new investigations, one of them is aged garlic extract (AG) which has been used for medicinal purposes since 3000 B.C. Recently, a clinical trial reported that 18 months use of AG reduced the periodontitis level as compared to the placebo group [21]. Garlic is well acknowledged for its dominant anti-inflammatory, antioxidant, antibacterial, antiviral, antifungal and antimutagenic properties [22,23]. These play a significant role in alleviating various oral diseases like pulpitis and periodontitis gingivitis, stomatitis, herpes labialis, oral candidiasis, dental plaque and oral cancers. It is a challenge for researchers worldwide to make proficient use of garlic and relish its maximum health benefits as it is the most inexpensive way to prevent or alleviate the effects of oral diseases.

**Figure 1:**
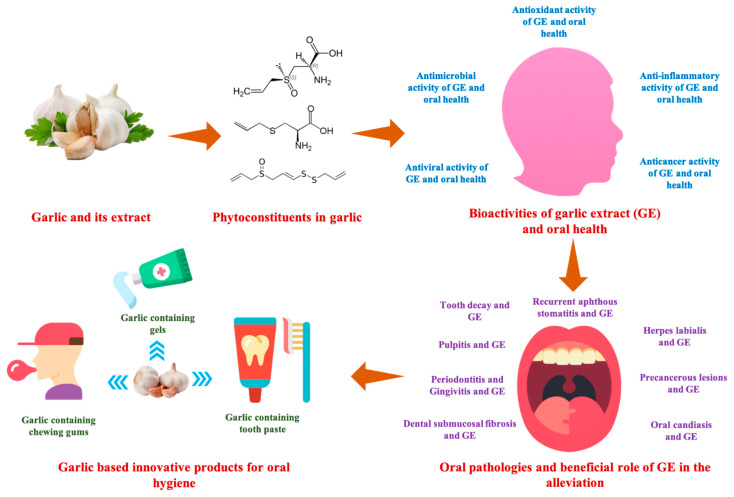
Various components discussed in the current review.

Many review articles have been explaining the bioactive compounds imparting therapeutic effects, whereas other articles with a dispersed content of literature on general health-promoting effects of garlic. There is no evaluative miscellanea on pivotal information on the role of garlic in the alleviation of dental disorders. Therefore, this current review will focus on the major bioactive compounds, potential pharmaceutical properties, and mechanism of action of GE in attenuating some of the oral pathologies. Various components discussed in the review are presented in Figure 1. 

## 2. Methodology

### 2.1. Selection Criteria

In the current study various bioactivities of garlic extract in alleviating oral pathologies have been reviewed by following the guidelines of ‘Preferred Reporting Items for Systematic Reviews and Meta Analyses’ (P.R.I.S.M.A. 2020) [24]. Studies selected to review were based on the following exclusion and inclusion criteria. Exclusion criteria included: (i) Studies that did not have full text available; (ii) in vitro, in vivo and clinical studies which does not follow ethical guidelines; (iii) published studies in local languages except English; (iv) studies examined other types of pathologies not related with oral pathologies.

Inclusion criteria included: (i) in vitro, in vivo and clinical studies with authentic data were included; (ii) studies published with English language were included; (iii) mostly studies from the period from 2010 to 2021 were reviewed; (iv) studies that examined effect of garlic extracts and its compounds on oral pathologies were selected.

The botanical name of garlic was followed according to the plant list database. For electronic literature search database such as Scopus, PubMed, Elsevier, Google scholar and Clinicaltrials.gov were used with following keywords on combination or alone i.e., oral pathology, garlic, phytoextract, cell cycle arrest, phytochemicals, bioactive compounds, antioxidant, antimicrobial, antiviral, anticancer, oral hygiene and oral microflora. The literature search was carried out from 12 April 2021 to 4 September 2021 and most of the studies reviewed were within the period of 2010 to 2021. A total of 210 studies were found from database in which using the exclusion criteria 19 duplicate studies and 13 studies having no full text were removed, leaving a total of 148 studies which were selected for review.

After the selection of in vivo, in vitro and clinical trial studies following inclusion criteria following data is collected: bioactive compounds found in garlic, various bioactivities of garlic extract against oral pathologies such as anti-viral, antifungal, antibacterial, antioxidant, anticancer and safety of garlic.

**Figure 2:**
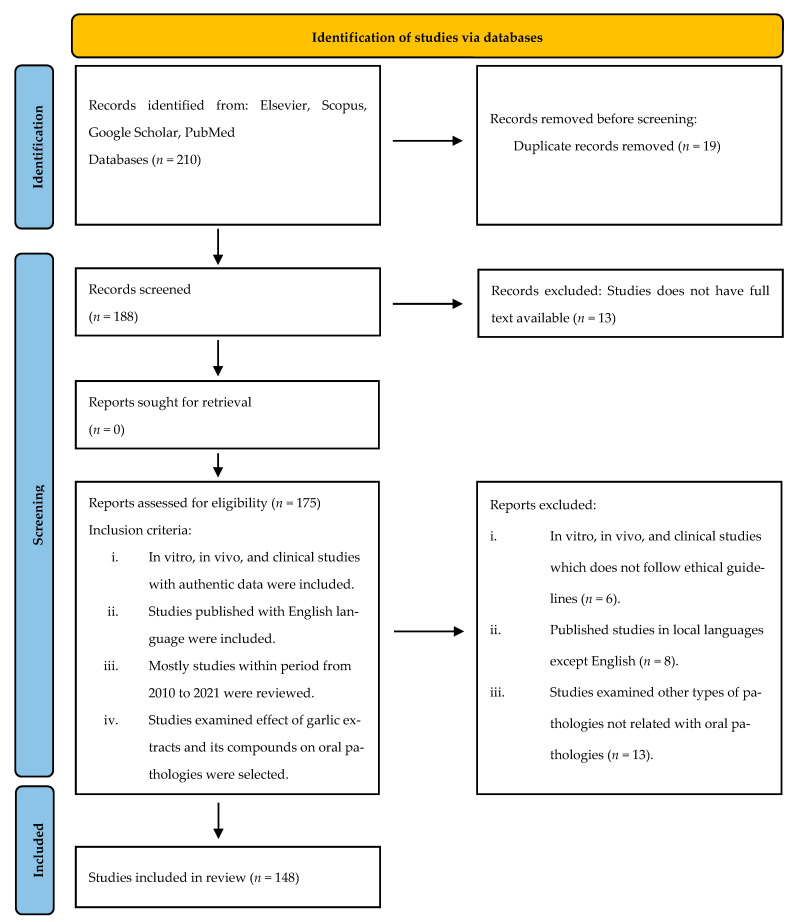
PRISMA flow diagram showing selection criteria.

The PRISMA flow diagram shows our selection process, the number of records identified and the eligibility criteria: exclusion and inclusion and number of studies reviewed in Figure 2.

### 2.2. Phytochemicals from Garlic with Relation to Oral Health

Garlic is one of the oldest common cultivated herbs. Several studies have reported garlic as an excellent source of diverse, active components which possess antioxidant, anti-inflammatory, antibacterial, antifungal, anticancer, cardiovascular protective, immunomodulatory, digestive system protective, anti-diabetic and anti-obesity like properties [25,26]. These bioactive compounds are mainly classified into organic sulfide compounds (OSCs), saponins, phenolic compounds and polysaccharides. Among them, organic sulfides are the most abundant bioactive compound in whole garlic. Fresh raw garlic bulbs contain protein—0.97 g/100 gW, carbohydrate (glucose—3.56, sucrose—0.12 g/100 g DW), dietary fibre (Neutral detergent fibre—9.97%, acid detergent fibre—9.09%, acid detergent lignin—3.92%, hemicellulose—0.47%, cellulose—2.07%); minerals (nitrogen 2.65%, carbon—41.28%, hydrogen—6.57%, sulfur—0.39%, phosphorus—1.18%, potassium—1.05%, calcium—0.22%, magnesium—0.06%); moisture content—63% and dry matter—39%. These active organosulphur comprises diallyl thiosulfonate (allicin), DAS, DADS, DATS, E/Z-ajoene, SAC, and S-allyl-cysteine sulfoxide (alliin) [27]. Allicin, i.e., diallyl thiosulfonate, is an active sulphur compound existing only in crushed raw garlic, whereas cooked garlic is devoid of this compound. Hence, it is suggested in traditional medicine to consume raw garlic for getting maximum oral and overall health benefits. 

The typical pungent flavour in garlic is due to allicin, which is also responsible for the burning and prickling sensations produced in the mouth by raw garlic [26,28]. Formation of allicin in garlic is achieved when enzyme alliinase acts upon odourless molecule alliin, i.e., S-allyl-cysteine sulfoxide, which account for almost 70% of total thiosulfinates present in crushed garlic cloves. Allicin is extremely unstable, which breaks down within hours at room temperature, and under a minute while cooking. On reduction, it produces various compounds DAS, DADS, DATS, ajoene, dithiins and allyl methyl trisulfide [28]. There are two general classifications of OSCs: oil soluble and water-soluble OSCs, whereas the former occurs in a more significant proportion [8]. SAC is a water-soluble, odourless OSC. SAC is highly stable in blood, so it is the most reliable detectable marker for garlic consumption [28]. Even after significant consumption of garlic, allicin, sulphides, ajoene, and other oil soluble OSCs cannot be identified in blood or urine [29]. Generally, OSCs are more digestible in raw garlic than in cooked garlic whereas saponin become more stable during the cooking process [27]. The most common examples of saponins found in Allium species are diosgenin, gitogenin and β-cholorogenin [30]. 

**Figure 3:**
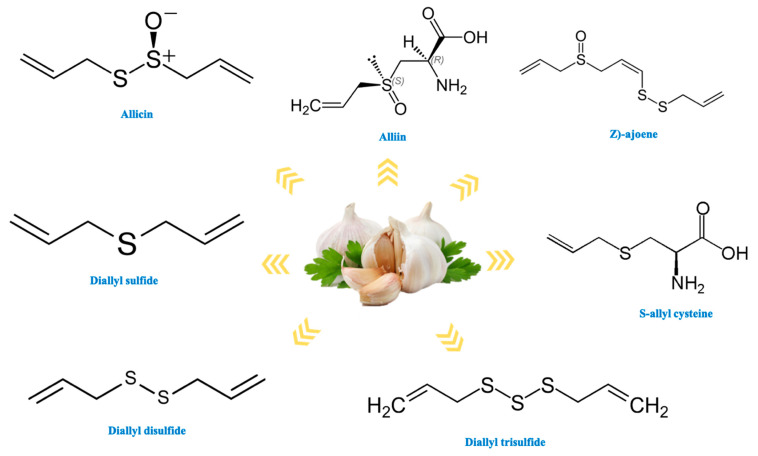
Structure of important bioactive constituents present in garlic.

In addition, garlic is rich in phenolic compounds, containing around 20, which is higher than many common vegetables. β-resorcylic acid is a major phenolic compound, followed by pyrogallol, gallic acid, rutin, protocatechuic acid and quercetin. Furthermore, garlic contains various sugars such as fructose, glucose and galactose [27]. Moreover, garlic is also a good source of vitamins such as ascorbic acid and B complex (B1, B2, B3, B5, B6, B9). Enzymes, arginine-rich proteins and minerals (calcium, iron magnesium, manganese, phosphorus, potassium, sodium, zinc, selenium) are other essential components present in garlic [26]. All these components of garlic are reported to alleviate one or more types of oral pathologies and improve oral health. Bioactive compounds present in garlic are presented in Table 1 and structures are shown in Figure 3. 

Black garlic has recently emerged to be rich in various bioactive components than the traditional varieties, which make black garlic more critical in managing oral health. Black garlic consists of prebiotic fibre, which, together with its medicinal value, makes it a potential therapeutic source in oral health. Dental plaque, periodontal disease, pulp disease and oral cancer are the most common diseases that have become a risk to oral health [32].

Black garlic (fermented white garlic) is a thermally processed product of fresh garlic without additives and transforms the garlic with reduced pungent odour and taste. In a recent in vitro study, it was reported that fermented black garlic extract (BGE) exhibits a strong killing effect on oral pathogen *Streptococcus mutans* and its multidrug-resistant isolates, however there is need to examine various activities of black garlic against oral pathologies, by further performing in vivo and clinical trial studies. During fermentation, its smelling lipid-soluble ingredients transformed into water-soluble components, which eventually will be discharged through the kidneys [33]. Throughout processing, in white garlic, the concentration of allicin decreases. Black garlic loses its noticeable flavour and allicin is transformed into antioxidant compounds such as SAC, bioactive alkaloids and flavonoids, which comparatively enhance the bioactivity in black garlic. This preparation of BGE makes it more potent and effective to be used in oral health care products [33]. 

### 2.3. Garlic Extract Preparation

There are two common types of GE: aqueous garlic extract (AGE) preparation and ethanolic garlic extract (EGE) preparation. In AGE preparation, the fresh garlic bulb with predefined weight is peeled and cleaned. Then evaporation of ethanol in a sterile laminar flow chamber is performed, followed by homogenization of garlic using a sterile mortar and pestle. Then, this homogenized mixture needs to be filtered through clean cheesecloth. This resulting extract will be of 100% concentration, and on further dilution, with appropriate volumes of sterile distilled water, the concentration of 75%, 50%, 25% and 10% can be made [34,35]. In EGE preparation, crushed garlic is moistened in 96% ethanol for 48 h. After that, the supernatant is to be filtered followed by rotatory evaporation at 40 °C. This extract should be then kept frozen at –20 °C. For the final concentration the frozen extract was reconstituted with normal saline [36]. 

Allicin is comparatively unstable and is rarely available for commercial purposes. It is either chemically synthesized by oxidation of the analogous polysulfides or purified from GE. There are some non-conventional techniques apart from conventional ones (AGE and EGE) to extract allicin, such as ultrasound-assisted extraction, pressurized liquid extraction, supercritical CO_2_ extraction and salting-out extraction based on different organic solvents, temperatures and sometimes enzymatic methods used for extraction. Whereas enzyme assisted subcritical water extraction has an advantage over these methods due to its use of water instead of organic solvent with a single step of the enzymatic extraction process [37].

### 2.4. Comparison of the Components of Fresh GE and BGE

The various components present in fresh garlic are transformed into a variety of bioactive compounds based on hydrolysis or oxidizing reaction. Compared with fresh garlic, water-soluble sugar, total polyphenols and flavonoids increase after processing for black garlic. Among the phenolic acids- caffeic acid, gallic acid and coumaric acid account for 99.3% in black garlic. The concentration of these compounds in fresh garlic accounted for 95.3% of the total phenolic content with lesser coumaric acid content. Furthermore, out of the total 17 OSCs identified, 64.2% were SACs derivatives, SAC (31%) and alliin (22.4%) were the major ones. Among the main γ-Glutamyl-*S*-Alk(en)yl-l-Cysteine derivatives, γ-Glutamyl-*S*-allyl-l-cysteine (17.5%) and γ-Glutamyl-S-allylmercaptocysteine (13.7%), accounting for around 31.1% of the total OSCs in the black garlic [38]. Some studies have reported about the increased concentration of amino acids like leucine (1.05-fold), isoleucine (1.68-fold) and phenylalanine (2.49-fold) after fermentation of white garlic to prepare black garlic. Some other compounds such as fructose and glucose and amino acids like cysteine (0.59-fold) and tyrosine (0.19-fold) decrease through the ageing process. Almost all the biological activities responsible for preventing and treating the oral pathologies are dependent on the antioxidant potential of the garlic extracts; hence, the potential of garlic antioxidant extracts are discussed in the following Section 3.1. 

## 3. Bioactivities of GE in Alleviating the Oral Pathologies

### 3.1. Antioxidant Activity of GE 

During the metabolic events, cells generate a variety of free radicals required for intracellular processes such as signal transduction, apoptosis, and proliferation. However, these free radicals are responsible for several ailments in the human body, such as neurological disorders, diabetes, cancer, ischemic diseases, inflammatory diseases, acquired immunodeficiency syndrome, hemochromatosis, emphysema and many others [39]. In recent years, there is an increasing consumer preference for using a natural source of antioxidants in nutraceutical pharmaceuticals and cosmeceutical industries [40]. Antioxidant compounds possess a significant antioxidant activity to minimize the adverse effects of free radicals.

Antioxidants present in garlic have been studied for their promising health-promoting activity against oxidative damage caused by ROS. HPLC profiling of GE depicted the presence of various OSCs such as allicin (1574.60–6771.03 μg/g), ajoene (41.60–644.20 μg/g), DAS (9.20–517.30 μg/g), DADS (4.80–92.70 μg/g), DATS (222.76–1324.30 μg/g), vinyldithiins 2-VD (31.20–2964.50 μg/g) which possess potent antioxidant activity [41]. They also reported TPC (2.43–11.21), DPPH (0.05–0.58 mg GAE/100 g DW), ABTS (0.02–164.80 mg GAE/100 g DW) and FRAP (12.30–164.80 mM TEAC/10 mg DW) in different garlic samples. Allicin, ajoene and 2-VD showed higher antioxidant activity compared to DAS, DADS and DATS. Oxidative stress is associated with periodontitis, a chronic inflammatory disease, triggered by bacterial infection that affects the regulation of the host inflammatory response [42]. There was lower salivary capacity and serum total antioxidant level in chronic periodontitis than in control individuals [43]. The malondialdehyde and 8-isoprostane; biomarkers of lipid peroxidation were higher in patients diagnosed with chronic periodontitis [44]. Particularly in the food industry, these compounds and their antioxidant potential are exhaustively evaluated because garlic can be used as an additive for delaying the formation of toxic oxidation products, controlling rancidity development, extending the shelf-life of products, and maintaining nutritional quality [45]. 

Similarly, another in vitro study reported TPC (1.48–3.48 mg GAE/g DW), DPPH (IC50: 6.25–33.28 mg/mL), ABTS (IC50: 11.46–46.53 mg/mL), and allicin content (127.33–165.43 mg/100 g DW) in lyophilized garlic powder [46]. Several reports indicated that oxidative stress is involved in oral lichen planus (OLP) pathogenesis. There was significantly higher salivary lipid peroxidation, ROS, nitrite and nitric oxide levels in patients with OLP than the control subjects [47]. Significantly lower total antioxidant activity was observed in OLP patients than in the healthy control group indicating the promising role of the oxidants to orchestrate via lipid peroxidation-mediated pathway [48]. The potential of antioxidant compounds from garlic in reducing the harmful effects caused by free radicals has been publicized by various studies. Hence, the utilization of garlic could be an alternative natural antioxidant-rich source to alleviate oral pathologies. 

### 3.2. Anti-Microbial Activity of GE

#### 3.2.1. Antibacterial Activity of GE

Many bacterial species are colonizing the human oral tract [49]. Bacterial adhesion to biomatter and their proficiency to generate biofilm on dentition are common steps in the pathogenesis of dental infections. Common oral pathogenic bacteria involved in the formation of dental caries include *Porphyromonas gingivalis*, *Staphylococcus aureus, Streptococcus sanguis, Streptococcus salivarius* and *Streptococcus mutans* [50]. These oral bacteria take in the carbohydrates from the food remnants present in our mouth and produce acids primarily lactic and acetic acids, as products of their fermentative metabolism. Furthermore, these acids cause the demineralization of hard tissue of teeth called enamel, which ultimately leads to cavity formation. Additionally, the inhabitation of certain Gram-negative anaerobic bacteria like *Aggregatibacter actinomycetemcomitans, Prevotella* sp. *Actinobacillus* sp. *Fusobacterium nucleatum* and *Porphyromonas gingivalis* in human gums result in periodontitis leading to swelling and damage in the connective tissue.

A myriad of antibacterial agents like cetylpyridinium chloride, chlorhexidine, amine fluorides and ethanol, which is frequently found in mouthwashes, have been utilized in the curing of oral bacterial infections. However, woefully, they have been shown to be toxic, stain teeth, or perhaps the reason for oral cancers and produces unpleasant taste [51]. Furthermore, increasing bacterial adaptation to antibiotics has led to the hunt for innocuous replaceable products such as natural pharmacologically active compounds isolated from plants [52]. A possible medicament to these oral bacterial infections can be offered by garlic. GE may prevent dental caries by promoting salivary secretion and repression of bacterial growth in the oral cavity. GE consists of specific bioactive compounds like alliin, allicin, SAC, DAS, allymethyltrisulphide and ajoene [53]. Specifically, allicin, also known as diallyl-thiosulfinate, is a class of organo-sulphur secondary metabolites immensely present in garlic with distinguished antibacterial properties [54,55]. The emergence of resistance against the allicin is also 1000 times slower than antibiotic resistance. Allicin acts essentially via impeding thiol group-containing enzymes, like alcohol dehydrogenases and cysteine proteases, vital for pathogens’ tissue damage and survival [10]. Allicin is secreted from the garlic cloves after tissue grinding by the action of the alliinase (a cysteine sulfoxide lyase) enzyme [56]. Furthermore, other thiosulfinates like allin (allyl thiosulfinate), ajoene, methyl allylthiosulfinates and propenyl allyl thiosulfinates are well-established quorum quenching molecules known to hamper bacterial growth because of their–S (O)-S- components that will interact with the sulfhydryl (SH) components of bacterial cell wall protein forming mixed disulfides [57]. In a randomized controlled clinical study, the oral intake of a few milligram concentrations of GE was shown to relieve both the gingival index (GI) and gingival bleeding index (GBI), pointing to GE can relieve the periodontal diseases as well [58]. In a recent in vitro study, the minimum inhibitory concentration (MIC) and minimum bactericidal concentration (MBC) of GE for the Gram-negative strains tested ranged from 35.8–1.2 mg/mL, MIC of pure allicin is approximately 4.2 mg/mL and allicin MBC was 7.8 mg/mL. These values were lesser than those for the Gram-positive strains where the garlic MIC ranged within 142.8–35.8 mg/mL, allicin MIC was approximately 27.4 mg/mL, and allicin MBC was 91.8 mg/mL [59]. Another mechanism of inhibition of biofilm formation is the twitching bacterial motility mechanism, which prevents bacterial colonization. In another in vitro study, GE was observed to inhibit *Streptococcus sanguinis* biofilm formation on hydroxyapatite discs model of the dental surface by intruding with bacterial motility mechanism using an optimal concentration of GE of 500 μg/mL. It is observed that these thiosulfinates in GE reacted with sulfhydryl components of *Streptococcus sanguinis* Tfp (Type IV pili) used for swimming motility, thus was impeded [60]. In a clinical study ethanol was identified to be the most promising solvent for GE, which showed more antibacterial activity with a 25 ± 2 mm zone of inhibition, followed by hexane extract with 19 ± 2 mm zone of inhibition on *Streptococcus aureus*. While diethyl ether GE was successful against *Streptococcus mutans* showed a 21 ± 3 mm zone of inhibition, and acetone GE showed an 18 ± 3 mm zone against this bacterium. Meanwhile, with different types of GEs, the MIC values ranged between 21 ± 3 mg/mL to 121 ± 7 mg/mL and MBC value ranged from 61 ± 6 mg/L to 214 ± 8 mg/mL. Moreover, they concluded that the antibacterial activity of GE is mainly due to the presence of phytochemicals such as tannins, flavonoids and alkaloids [61]. To conclude, GE bulbs can be effectively used to treat periodontal and dental caries infections. However, care must be taken while directly taking garlic as there is a risk of mucosal damage by the GE [62]. 

#### 3.2.2. Antifungal Activity of GE

As established by several experimental research, GE is regarded as one of the most prominent medicinal herbs with extensive antimicrobial properties and a viable treatment choice for a myriad of oral diseases, including fungal infections. The most prevalent fungal infection of the oral mucosa is incited by species of the genus *Candida* among which *Candida albicans* is the most aggressive pathogenic *Candida* species. However, several other species of *Candida spp.,* like C. *albicans*, *C. glabrata*, *C. tropicalis*, *C. dubliniensis*, *C. krusei* etc., are also responsible for oral mycoses, i.e., Candidiasis. Likewise, *Aspergillus fumigates* is responsible for Aspergillosis, *Blastomyces dermatitidis* for Blastomycosis, *Coccidioides immitis* for Coccidioidomycosis, *Cryptococcus neoformans* for Cryptococcosis, *Fusarium moniliforme* for Fusariosis, *Geotrichum candidum* for Geotrichosis, *Histoplasma* *capsulatum* for Histoplasmosis, *Mucorales* for Mucormycosis, *Paracoccidioides brasiliensis* for Paracoccidiomycosis, *Penicillium marneffei* for Penicilliosis, *Sporothrix schencki* for Sporotrichosis [63]. GE have a robust antifungal effect and inhibit mycotoxins formation. Allicin (diallyl thiosulphonate) was assumed to be the main component among several bioactive compounds responsible for inhibiting fungal growth [64]. The enzyme alliinase converts alliin into allicin when garlic is cut or crushed [65]. Allicin is capable of transmembrane movement to combine with sulfur-containing molecular groups in proteins. As a result, glutathione is oxidized, resulting in the activation of microbial apoptosis. The antifungal effect of GE is due to the inhibitory function of allicin (the active component of garlic) on thiol enzymes [66].

The efficacy of garlic paste in oral candidiasis was examined in human clinical study (randomized clinical trial) and it was concluded that topical use of garlic paste for 14 days was more advantageous than that of clotrimazole solution in suppressing the clinical signs of oral candidiasis and producing successful clinical outcomes [67]. Thus, garlic could be a good option for the treatment of oral candidiasis. While comparing the therapeutic effect of AGE and nystatin mouthwash against denture stomatitis in randomized clinical trial it was reported that the mean width and length of erythema under the denture after three weeks of treatment was 0.08 ± 0.18 cm and 0.11 ± 0.21 cm, respectively, in the nystatin group and 1.09 ± 0.5 cm and 0.99 ± 0.34 cm in GE group (at the start of treatment, the mean width and length of erythema in nystatin group was 3.03 ± 1.03 and 3.61 ± 0.88 cm, respectively, and in garlic, it was 3.63 ± 1.21 cm and 3.53 ± 1.16 cm, respectively). Though Nystatin mouthwash was found to produce faster recovery of lesions compared to GE, it was less preferred because of its side effects like nausea, vomiting, diarrhea, anorexia etc., as well as having a bitter taste. Thus, GE could be regarded as a better alternative in treating denture stomatitis [68]. Likewise, in vitro study the antifungal effect of AGE on *C. albicans* was investigated and they found that it was able to inhibit the growth of *C. albicans,* but its effect was less than nystatin [69].

Similarly, the antimicrobial efficacy of garlic with lime against *C. albicans* was proved in randomized double-blinded controlled clinical trial as the *C*. *albicans* count reduced from 7.1 × 10^4^ to 4.3 × 10^4^ CFU/mL after two weeks of rinsing [66]. In another in vitro experiment, the efficacy of three different concentrations (10, 20, and 30%) of garlic and propolis extract against *C. albicans* is examined and compared it with amphotericin-B 10 mg (control) at 24 and 48 h. They did not find a significant difference in inhibition of candidal growth at 10% concentration. However, at 20 and 30% concentrations, complete inhibition of the growth of *C. albicans* with GE was evident at both 24 and 48 h. Propolis could not inhibit the candidal growth with 10 and 20% concentration at 24 and 48 h, but it showed inhibition at 30% concentration at 24 h duration [70]. Thus, it was concluded that GE could be a potential agent in eradicating *C. albicans* in chronic periodontitis patients. Clinical study on short term effect of heat-killed *Lactobacillus acidophilus* (LF) and GE on *C. albicans* resulted in the reduction in the total erythematous area dimension to less than 2 mm for both LF and GE from 3 ± 0.40 mm for LF and 3.20 ± 0.54 mm for GE whereas, for the control (essential standard management only), it was reduced to 2.69 ± 0.43 mm from 3.11 ± 0.64 mm. Furthermore, the *C. albicans* counts were 1.89 ± 0.928 for LF, which was initially 48 ± 3.202 and 3.33 ± 1.14 for GE, which was initially 48.78 ± 2.99, while for control, it was 42 ± 4.272, which was initially 49 ± 3.87. Similarly, *C. albicans* biofilm-forming activity was 0.26 ± 0.07 and 0.31 ± 0.08 for LF and GE, respectively, whereas for the control, it was 5 ± 0.43 [65]. A recent in vitro study reported the essential oil of GE was more effective than fluconazole in inhibiting both planktonic cells in vitro and biofilms of different *Candida species* (*C. albicans*, *C. glabrata*, *C. tropicalis* and *C. krusei*) isolated from dental prostheses while studying the antifungal activities of essential oil of GE and fluconazole against clinical isolates of *Candida species* [71]. An in vitro study to analyse the activities of garlic essential oil against three *Candida species* (*C. albicans*, *C. glabrata* and *C. tropicalis*) showed the lowest value (0.4 µg/mL) of MIC for *C. albicans* followed by *C. tropicalis* (0.5 µg/mL) and *C. glabrata* (0.6 µg/mL). Similarly, the Minimum Fungicidal Concentration (MFC) was also lowest for *C. albicans* (0.7 µg/mL) whereas, for both *C. tropicalis* and *C. glabrata*, the MFC value was found to be 0.8 µg/mL, which leads to the conclusion that garlic essential oil was effective in the inhibition of these fungi [72].

Since garlic is non-chemical, non-synthetic, has no adverse effects and is also a rich source of several bioactive compounds, including allicin, it can be used as a good alternative in alleviating different oral fungal pathologies.

### 3.3. Antiviral Activity of GE

Several DNA and RNA viruses are responsible for mild to severe diseases in the human body. Numerous viruses have been reported in the oral cavity that causes infection in the mucosal epithelium, which may lead to ulceration or blistering in the oral tissue [73]. Primarily the oral infection is caused by the members of human herpes virus (HHV) (including herpes simplex virus (HSV-1 and 2), varicella-zoster virus (VZV), Epstein-Barr virus (EBV), cytomegalovirus (CMV), HHV-6, HHV-7, and HHV-8) and human papillomaviruses (HPV), which cause diseases like herpes ulcers, precancerous lesions, herpes chicken pox, enanthem, tumours, herpes zoster, periodontitis, condylomas, papilloma and focal epithelial hyperplasia, nasopharyngeal carcinoma and are also associated with oral cancers. Furthermore, secondary pathological processes may also affect the oral mucosa [74,75]. Moreover, the association of severe acute respiratory syndrome coronavirus 2 (SARS-CoV-2) could be directly or indirectly related to the lesions of the oral mucosa; however, this is still unclear [76]. 

However, minimal effective treatments are present for viral infection because many existing antiviral drugs have limitations like toxic side effects, drug resistance, and poor bioavailability. Therefore, there is always a need for alternatives such as synthetic chemical compounds or natural products’ compounds. Garlic is rich in various phytochemicals, especially allicin, with a substantial anti-microbial potential (Section 3.2). Allyl methyl sulphide is one of the primary active compounds of allicin, which interact with the viral phospholipids and amino acids involved in infection and prevents them from attachment to the host cell by denaturing these viruses [77]. However, very few reports are available related to garlic in alleviating oral diseases. 

A commercial GE was obtained from a factory in Shanghai and tested for antiviral activity against HSV-1. In an in vitro experiment, the virus was grown in rabbit skin cells, evaluated using plaque counts, and treated with 0–1.5 mg/mL. The concentration of 0.015 mg/mL or higher was reported for significant effect against HSV-1 during the incubation period [78]. In another in vitro experiment, the virucidal effects of GE were extensively studied and evaluated the anti-viral effect of the fresh extract, polar fraction and garlic associated compound against HSV-1, 2 and other viruses. The fresh GE (8–1000 mg/mL) exhibited a virucidal effect against HSV-1 and -2 in the concentration-dependent manner. However, in the cytotoxicity assays, it was found toxic against the HeLa and Vero cells at the concentration of 11 and 3.5 mg/mL. The active compounds of fresh extract are identified as thiosulfinates that contains diallyl thiosulfinate (allicin) (2.5 mg/mL, ailyl and aliyl methyl thiosulfinates (0.63 mg/mL) and trans-1-propenyl allyl thiosulfinate (0.23 mg/mL). Direct virucidal activity and significant cytotoxicity were reported for allicin, but no intracellular antiviral properties were observed. The overall antiviral activity was due to the presence of allicin. HSV-1 and HSV-2 both were highly sensitive to the allicin. However, the sensitivity also depends on the nature of the viral envelope than on nucleic acid type [79].

**Figure 4:**
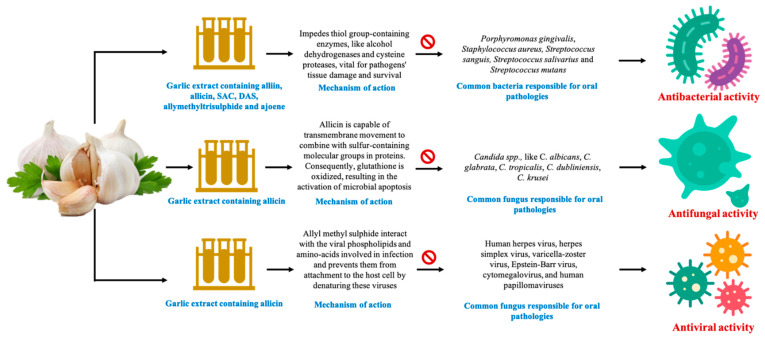
Antimicrobial and antiviral activities of garlic extract against oral pathogens.

An in vitro study evaluated the antiviral activity of garlic essential oil of under cytopathicity assay using virus-infected African green monkey kidney (Vero) cell line (grown in 96 well plates and infected with 100 µL of stock virus) against Herpes Simplex Virus-1 (HSV-1) at the concentration of 200, 500, and 1000 µg/mL (prepared in DMSO). The results showed that the antiviral activity was highest with the concentration of 1000 µg/mL, and it was in a concentration-dependent manner (37.66, 72.94, and 93.81%), and the treatment significantly improved the viability of treated cells as compared to control. The effective concentration (EC_50_) value was 320 µg/mL. The author concluded the antiviral activity was attributed to the chemical constituents of GE specially di-2-propenyl disulfide, methyl-2-propenyl trisulfide and di-2-propenyl trisulfide [80]. In another in vitro trial, aqueous and alcoholic garlic extract was used against HSV-1 collected from the infected patients. The study reported that 2.9 mg/mL and 3.05 mg/mL of aqueous and alcoholic extract respectively causes 50% cell death in cell culture (CC_50_) due to cytotoxic effect. However, the IC_50_ value was 1.13 and 0.98 mg/mL for aqueous and alcoholic extracts, respectively. The author found both the aqueous and alcoholic extract were effective against HSV-1, but there was a requirement for a higher dose for cytotoxic effect than anti-viral effect [81]. The findings suggested that the application of GE is beneficial to treat oral viral infections. Antibacterial, antifungal, and antiviral activities of garlic extract against oral pathogens are shown in Figure 4. 

### 3.4. Anti-Inflammatory Activity of GE

Inflammation is a defence response in the human body that occur as a result of harmful stimuli. One major class of bioactive compounds derived from the compound alliin are organic sulphurs such as SAC, SAMC, and N-acetylcysteine. SAC possess anti-inflammatory, anti-apoptotic and antioxidant properties, while SAMC possesses anti-cancerous activity [25]. Organo sulphur compounds such as thiacremonone extracted from garlic have anti-inflammatory effect by inhibiting nuclear factor-kB (NF-κB). NF-κB act as one of the important target molecule of organo sulphur compounds from garlic and it is also implicated as transcription factor that regulates genes responsible for the inflammatory responses [82]. In one of the studies, GE significantly inhibited damage caused by *Eimeria papillate* infections and inflammation in liver [23]. 

Oral diseases such as gingivitis, periodontitis, oral cancer, receding gums and plaque margin accumulation due to infection caused by gram-negative and -positive bacteria are major health concerns due to their high prevalence in all regions of world. Adverse oral health affects the quality of life, economic productivity and systemic health. More than 10% of expenditure is related to curing oral diseases in developed countries [61]. Traditional plants and their natural phytochemicals can be used to treat oral health problems as the best alternative to synthetic chemicals. Garlic is one of the most extensively used medicinal plants for treating various diseases related to oral health [10]. Aged garlic extract prepared by ageing garlic for >10 months in ethanol found to contains pharmacologically active sulfur-containing amino acids, such as SAC, S-1-propenylcysteine (SIPC), SAMC and DAS. It was demonstrated that DAS reduces inflammatory reactions elicited by *Porphyromonas gingivalis* derived lipopolysaccharide (LPS) in human gingival fibroblast cells and inhibits the growth of periodontal microbials [83]. A recent in vivo study reported that GE prevents the cells from different phases of cancer by neutralizing the free radicals, increasing the actions of antioxidant enzymes such as glutathione S-transferase and catalase, avoiding chromosomal damage, DNA repair mechanisms and improving glutathione contents [84]. Garlic and its oil-soluble compounds viz. DADS reduces the activation of carcinogens by arresting the G2/M stage of the cell cycle, which further enhances phase 2 detoxifying processes and increases acetylation of histones and encourages mitochondrial apoptotic pathway. They also inhibit the propagation of tumour cells in vitro through the induction of apoptosis [85]. A randomized clinical trial study found that an oral intake of AGE tablets (300 mg AGE powder) for 18 months was an effective supplement for preventing and improving periodontal disease, a chronic inflammation resulting from progressive detachment of gum tissue from the tooth [86]. Clinical studies also suggested that daily consumption of AGE for at least four months could reduce gingival inflammation and bleeding [58].

#### Mechanism of Modulation of Immunomodulatory Factors like TNF-α, IL by GE in Oral Diseases

Much evidence is based on in vivo and in vitro investigations of garlic showing significant effects on the immune system. Bioactive compounds derived from GE inhibits the transcription of several cytokine genes such as tumour necrosis factor-α (TNF-α), interleukin-1beta (IL-1β), IL-6, monocyte chemoattractant protein-1 (MCP-1), and IL-12 involved in proinflammatory responses [87]. In one in vitro study, AGE up regulated the production of IL-10 and decreased IL-12 which further downregulated the proinflammatory cytokines TNF-α, IL-6, and IL-2 by T cells, thus acting as a negative feedback in the signalling of proinflammatory response [88]. Garlic and its associated bioactive compounds exert stimulatory and inhibitory effects on whole blood cultures of monocytes and lymphocyte proliferation which controls the proinflammatory cytokines by TNF-α generation and IL-10 production [88,89]. Garlic bioactive compound 1,2-vinyldithiin attenuates the secretion of IL-6 and MCP-1, -2 in human preadipocytes treated with macrophagic factors. Both molecules are related with metabolic complications of obesity and inflammation [89]. Hence, it could be suggested that GE possess several bioactive compounds which have great potential in reducing oral health problems. 

### 3.5. Anti-Cancer Activity of GE

**Figure 5:**
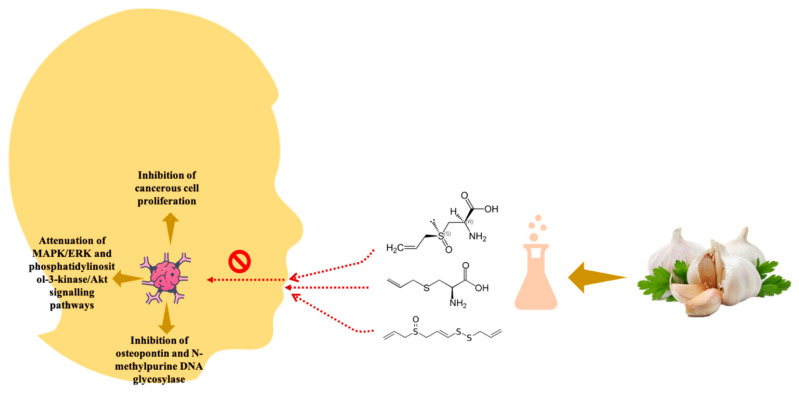
Anticancer activity of GE.

GE and formulation are widely studied for their anti-carcinogenic potential and reported to be involved in detoxifying carcinogen, suppressing cell proliferation and growth, cell invasion, suppression of metastasis, and immunomodulation of tumour cells [8]. The sulphuric compounds, which constitute ~2.3% total weight of garlic, have shown positive effects in managing various cancers [90]. Many in vitro and in vivo findings demonstrated that GE kills cancer cells by acting as a free radical scavenger, suppressing mutagenesis, and modulating the cancer cell behaviour, i.e., proliferation or migration and promoting apoptosis in cancerous cells [8]. Water-soluble (alliin, cysteine, SAC, SAMC) oil-soluble sulfur compounds (allicin, ajoene, DAS, DADS, and DATS) have been positively correlated with the anticancer effect of GE. More specifically, GE has been reported to be an effective solution for oral cancer. A recent in vitro study reported that SAC shows an inhibitory effect on the human oral cancer cell line (CAL-27). The authors showed that SAC could stabilise the E-cadherin/β-catenin complex in oral cancer cell lines, whereas structural and functional deregulation of β-catenin and E-cadherin is positively correlated to oral cancer progression. The authors concluded that SAC inhibits the mitogen-activated protein kinases/extracellular signal-regulated kinases (MAPK/ERK) signalling pathway and down-regulates the SLUG repressor protein, ultimately showing the anti-oral cancer effect [91]. SAC regulates the E-cadherin expression and attenuates the progression of malignant of oral cancer in human. Allicin also inhibits Ornithine decarboxylase enzyme activity involved in polyamine biosynthesis, reducing cellular polyamine levels and inhibiting cell proliferation and apoptosis. In another in vitro study, titanium oxide (TiO_2_) nanoparticles demonstrated relatively higher cell viability in oral cancer (KB) cell lines, whereas modified TiO_2_ nanoparticles with natural GE showed a more prominent anticancer effect on KB cell lines. The authors demonstrated that 10 mg/mL of garlic modified TiO_2_ nanoparticles showed 60.76% of cell viability and concluded that the high antioxidant activity of GE resulted in increased anti-oral cancer activity [92]. Another in vivo study reported the anti-oral cancer effect of GE in Syrian hamsters [93]. In another investigation, the anti-oral cancer effect of SAC was studied in vivo in the mouse model [94]. The consumption of SAC significantly reduced the progression of oral cancer in mice. The consumption of five and 40 mg SAC/kg body weight reduced the tumour volume from 177 to 125 mm^3^ and 177 to 47 mm^3^, respectively, after the 4th week. It is reported that SAC suppresses the factors related to oral carcinogenesis, i.e., osteopontin and N-methylpurine DNA glycosylase. Osteopontin is a non-collagenous, sialic acid-rich glyco-phosphoprotein involved in the development and remodelling of bone tissue and is recognised as a prognostic factor for oral tumour progression [95]. The chemo-protective effect of SAC is associated with suppressing OPN and DNA glycosylase. SAC reported to interplay with several proteins involved in cell-to-cell recognition, signal transduction, effector proteins regulating the oral tumour growth and proliferation [96]. The molecular analysis reflected that SAC attenuates MAPK/ERK and phosphatidylinositol-3-kinase/Akt signalling pathways in tumour-bearing mice Figure 5.

Garlic phytochemicals induce the phase II detoxification enzymes such as, epoxide hydrolase, glutathione-s-transferases, quinone oxidoreductase and glucuronosyl-transferases involved in modifying carcinogens facilitating their excretion. Oxidative stress mediates lipid peroxidation, and free radical production sets up the carcinogenesis process. The antioxidant potential of GE helps lower oxidative stress and thus suppresses carcinogenesis [97]. 

It is evident from the vast number of studies that GE and bioactive components present in it can act as a natural remedy in managing the discomfort caused by oral cancer and find applications in the pharmaceutical and functional food industry. Table 2 shows relevant case studies showing the effect of bioactive garlic compounds in the management of oral cancer.

## 4. Oral Pathologies and Beneficial Role of GE in the Alleviation 

Tooth decay, periodontal, pulpitis, recurrent aphthous stomatitis, herpes labialis, precancerous lesions, dental submucosal fibrosis, oral candidiasis, OLP and halitosis are the common oral pathologies caused by inadequate oral hygiene and an unhealthy lifestyle. Allicin, DADS, DATS, methyl allyl thiosulfinate allyl methyl thiosulfinate, deoxyalliin, ajoene and alliin bioactive compounds found in garlic, can reduce the inflammatory response, reduce neutrophil migration, inhibit bacteria and viruses, oppose oxidation and improve inherent immunity [25]. The effectiveness of GE in the management of various oral pathologies are discussed in the following sub-sections. 

### 4.1. Tooth Decay

Acid production owing to carbohydrate fermentation by *Streptococcus mutans*, *Streptococcus sobrinus*, and *Lactobacilli* causes tooth decay or dental decay. In recent in vitro study fresh soft neck and stiff neck garlic species were extracted with distilled water, and it was discovered that the bioactive component allyl 2 propenethiosulfinate or diallyl thiosulfinate present in GE has antibacterial activity against the cariogenic bacteria *Lactobacillus acidophilus* (MTCC 10307) and *Streptococcus mutans* (MTCC 497) with the greatest zone of inhibition of 24 mm. As a result, it may be a valuable resource for treating dental caries and other oral illnesses [104]. 

### 4.2. Pulpitis and GE

Pulpitis is a condition in which the dental pulp is inflamed due to the microleakage of bacteria, causing the pulp to enlarge. GE as an antibacterial agent can diffuse inorganic tissue with minimum toxicity. In recent ex vivo study the efficacy of sodium hypochlorite (NaOCl) and garlic in smear layer removal in root canals is examined. They observed the elimination of irrigant as well as debris from root canals in 68 single-rooted mandibular premolars treated with 5% NaOCl and 17% ethylenediaminetetraacetic acid (EDTA) with GE (64 mg/mL) prepared by garlic cloves in 70% (*v*/*v*) ethanol [105]. 

In a human clinical study, 30 children (ages 7 to 9) have bilateral primary teeth indicated for orthodontic serial extraction were distributed into two groups: group I (15 children: right side tooth treated with tri-antibiotic paste (group IA), left side tooth treated with formacresol (group IB), and group II (15 children: right side tooth treated with *Allium sativum* oil (group IIA) and left side tooth treated with formacresol (group IIB). After 15 days, in groups IB, IIB almost 33.4% of cases had unadorned inflammation, whereas 66.7% had moderate inflammatory infiltration with lymphocytes and macrophages. After 30 days, approximately 86.7% of cases in group IB had moderate, 6.7% mild, and 6.7% severe vascularity of pulp tissue. There was a significant difference in pulpal inflammation, vascularity and fibrosis between groups treated with formocresol, triantibiotic paste and *Allium sativum* oil [106].

### 4.3. Periodontitis and Gingivitis and GE

Periodontal disease, often known as gum disease, is caused by microbes such as *Treponema denticola*, *Porphyromonas gingivalis*, *Actinobacillus actinomycetemcomitans*, which cause tooth loss. A recent in vitro study reported that with a MIC of 16.6 μL/mL, AGE showed more robust bacteriostatic activity against *Porphyromonas gingivalis*, and the gelatin liquefaction was modestly decreased by 250 μL/mL dose of AGE, indicating that garlic has antiproteolytic effect against *Porphyromonas gingivalis* protease [49]. In another in vitro study, garlic extract (142.7 mg/mL garlic with 55 µg/mL allicin) inhibited *Porphyromonas gingivalis* trypsin-like and total protease activity by 92.7 and 94.88%, respectively, with minimal inhibitory concentrations (4.4 mg/mL garlic) and MBC (8.9 mg/mL garlic) [59]. In a randomized controlled double-blind clinical study, the efficacy and impact of AGE on periodontitis in humans is investigated. 201 healthy adults were chosen for this human clinical study, ranging in age from old to severe (minimum of three periodontal sites). The control group was instructed to take AGE extract tablets for 18 months. After 10 months, the mean value of pocket depth for AGE was 1.06 0.49, compared to the baseline value of 1.50 0.46, indicating that AGE can prevent or improve periodontitis. As a result, bioactive compounds in garlic appear to suppress the growth of oral infections and some proteases, which is particularly important for treating periodontitis patients [86].

Gingivitis is the prolonged inflammation of gingival tissue caused by anaerobic bacteria colonized on the dental plaque. In neglected conditions, chronic inflammation leads to resorption of alveolar bone, periodontitis and loss of the tooth. Prevention or removal of dental plaque requires mechanical removal or clinical treatment with antibiotics [83]. Several indices such as GI, Modified GI, Sulcus Bleeding indices, Plaque indices have been developed primarily by correlating the visual clinic symptoms with histologically determined inflammation of gingivae [107]. Subgingival irrigation with a herbal-based mouth rinse effectively reduces the Sulcus Bleeding indices and GI, recommended for an adjunctive procedure for reducing the gingival inflammation [108]. *Salvadora persica* ethanol extract and Aloe vera gel exhibited higher efficacy than most widely used chlorohexidine against cariogenic bacteria [109]. A randomized controlled, double-blind clinical study demonstrated that AGE effectively prevents periodontal disease [86].

### 4.4. Recurrent Aphthous Stomatitis and GE

The most prevalent multifactorial T cell-mediated immune-dysregulated disease is recurrent aphthous stomatitis, linked to various bacterial and viral infections, allergies and hormonal imbalance [110]. Forty-two patients were investigated in a clinical trial, patients with minor recurrent aphthous stomatitis were split into groups: Group A was administered with 5 mg allicin/5 mL mouth rinse whereas group B was administered with 250 mg capsules of allicin). Results showed significant decrease in the ulcer size on 7th day of administration with pain score of zero, and 7, the mean erythema score of 0.16 ± 0.38, and zero, respectively for group A and B [111]. By downregulating IL-5 beta mRNA levels, allicin can reduce the concentration of inflammatory mediators such as TNF-alpha, interleukins, and interferon-gamma by peripheral blood mononuclear cells [112].

### 4.5. Herpes Labialis and GE

Herpes labialis (cold sores), is caused by the herpes simplex virus type 1 (HSV-1) and manifests itself as searing pain with tiny blisters or sores, fever and lymph node enlargement. According to recent clinical study, aged garlic extract inhibits, DNA synthesis, reverse transcriptase, and viral RNA polymerase as well as decrease the expression of the extracellular signal-regulated kinase/mitogen-activated protein kinase signalling pathway [113]. A recent in vitro study shows, when the African green monkey kidney (Vero) cell line (virus-infected cells) was treated with garlic essential oil (EC_50_ 320 μg/mL), which contains dimethyl trisulfide, limonene, diallyl tetrasulfide, diallyl pentasulfide and methyl-trans-propenyl-disulfide bioactive compounds, showed maximum % inhibition of DPPH (87 ± 1.65) at 200 µg/mL concentration and antiviral activity (93.81 ± 2.06) at 1000 µg/mL concentration than untreated cells [80].

### 4.6. Precancerous Lesions and GE 

Chewing areca and smoking tobacco causes a precancerous lesion that increases the risk of mouth cancer. Diffuse scaling, thicker epithelium with microscopic greyish-white plaques, inflammatory regions and linear fissures are all common symptoms of this disease. In recent human clinical trial, for one month ten patients with potentially malignant oral dysplastic diseases were given topical SAC; a common bioactive compound of aged garlic extract with Orabase at a concentration of 5 mg SAC/1 g Orabase. At the end of the month, they found that treatment effectively reduced lesions’ size (15.06%), discomfort, pain score and histological improvement compared to untreated patients [114].

### 4.7. Dental Submucosal Fibrosis and GE

Dental submucous fibrosis is a well-known, possibly malignant oral condition that results from a juxta-epithelial inflammatory reaction and escalating fibrosis of the submucosal tissue, causing collagen metabolism disturbance. In recent human clinical trial, 26 patients of stage II oral submucous fibrosis received an intralesional injection of thio-2-propene-1-sulphinic acid S-allyl ester for sixteen weeks, at 40 weeks, the net gain in mouth openness was 5.16 ± 1.04 mm, burning sensation improved by 4.33 ± 1.04, and the oral health impact profile-14 score improved by 12.589 ± 82 [115]. In another human clinical trial, 15 patients with oral submucosal fibrosis were given pentoxifylline (400 mg) for three months with garlic pearls thrice daily. Patients who were treated had a 95.68% lessening in burning sensation and a 5.38 mm upsurge in the mouth opening [116].

### 4.8. Oral Candiasis and GE

Oral candidiasis is a fungal infection that affects the oral mucosa and causes whitish or yellowish sores. *Candida albicans* causes diabetes, Cushing’s syndrome, cancers and immunosuppressive diseases by affecting salivary gland function [100]. A recent in vitro study reported, alliin significantly reduced fungal growth, a bioactive component present in garlic essential oil (30 μL). With the lowest MIC value (0.4 μg/mL), *Candida albicans* (PTCC 5027) was the most vulnerable *Candida* spp. to garlic essential oil, followed by *Candida tropicalis* (ATCC 13803) (0.5 μg/mL) and *Candida glabrata* (PTCC 5297) (0.6 μg/mL) [72]. Bioactives in garlic essential oil, such as diallyl sulphide and methyl allyl disulfide, act as antifungal agents, causing cytoplasmic leakage by rupturing the outer layer of fungal and bacterial liposaccharides. 

It is evident from the findings by various researchers that GE is very effective in alleviating the major oral pathologies. Table 3 presents the role of GE in the alleviation of oral pathologies.

### 4.9. Dental Plaque and Anti-Biofilm Potential

Although dental plaque is preventable but is a significant cause of dental caries, periodontitis and other associated complexities, it is an extracellular matrix embedded with bacterial microflora of host or microbial origin, providing conditions for proliferation and colonization of dental bacteria and fungi [124,125]. The antibacterial potential of GE has also been demonstrated against dental plaque bacteria. A recent in vitro study reported that GE is sensitive to dental bacteria *Streptococcus mutans, S. sanguis, Lactobacillus spp. S. salivarius,* and *Pseudomonas aeruginosa* and recommended its use in toothpaste and mouth wash [126]. The anti-biofilm potential of GE was also evaluated on orthodontic mini-plants using microbial visibility assay, electron dispersive X-ray spectroscopy and Scanning electron spectroscopy analysis and suggested as a promising alternative to the prevailing synthetic agent [127]. In recent in vitro study, GE loaded sol-gel-based nanoparticles exhibited significant enhancement in antimicrobial and anti-biofilm activity against well-established biofilms of methicillin-resistant *Staphylococcus aureus* [128]. In another in vitro study the antibacterial efficacy of two commercial root canal sealers, MTA fillapex and gutta-flow 2 is examined and result shows that garlic and chitosan incorporation significantly improved its antibacterial efficacy against *E. faecalis* [129]. Similarly, Garlic oil was also found effective in sterilization of K-file used in endodontic treatment [130].

### 4.10. Oral Microflora and Antibiosis

Bacteria find a predominant place in Oral microflora and causes dental caries, periodontitis, gingivitis, tooth decay and ultimately tooth loss [131]. Although synthetic agents’ chlorohexidine and antibiotics are widely used clinical solutions, associated side effects and rising antibiotic resistance have become serious health concerns [132]. Plant/herbal formulations are considered a safer alternative to this problem. Antibacterial use of garlic has been studied extensively, and a vast spectrum of bacterial species found sensitive to garlic extract. A recent in vitro study reported, AGE to be effective against periodontal microbes, *Aggregatibacter actinomycetemcomitans, Fusobacterium nucleatum* and *Porphyromonas gingivalis* with MIC of 4.3 mg/mL, 1.1 mg/mL and 17.2 mg/mL, respectively [133]. Another in vitro study reported sensitivity of 70 bacterial strains to GE with a minimal inhibitory concentration of 8 µg/mL. These strains were moderate to highly resistant to erythromycin and or methicillin [134].

Polyphenols also limit bacterial growth and adhesion, inhibiting proteolytic enzymes either by direct biosynthesis or interfering with the action. Gingipains are produced by bacteria such as *P. gingivalis* in the dysbiotic oral ecosystem, trypsin like cysteine caspases are involved in many proteolytic degradation of many immune components, including antibacterial peptide defensins. Polyphenols also modulate local oral immunity by decreasing the pro-inflammatory factor, IL-1β (Interleukin-1b), IL-5, IL-8, COX-2 (Cyclooxygenase-2), TNF-α and increases anti-inflammatory components [118]. 

Diallylpolysulfanes of distilled garlic extract reacts with metabolites of low molecular weight (glutathione), which plays a vital role in managing oxidative stress. Additionally, many vital proteins with exposed cysteine residues undergo thiol-polysulfane exchange reaction, leading to activity loss [135].

## 5. Garlic Based Innovative Products for Oral Hygiene

### 5.1. Chewing Gum

Chewing gums are a soft, insoluble, cohesive confectionary product chewed for its various intended purpose. The purpose of chewing gums has diversified from a flavouring confectionary to a drug delivery system. Medicated chewing gums are loaded with bioactive compounds, including nutraceuticals or medicine for targeted health benefits. Chlorohexidine acetate has been successfully used in chewing gum-based delivery systems as adjuncts in clinical treatment [136]. Volatile sulfur compounds are present in many plant edible parts, including garlic or produced by gram-negative bacteria (anaerobic) associated with periodontitis or tongue that produces halitosis or malodor [137]. Chewing gum loaded with magnolia bark extract and zinc acetate effectively reduces volatile Sulphur compounds responsible for bad breath or halitosis [138]. Sugar-free chewing gums also reduces halitosis by interacting with volatile sulfur compounds or responsible bacteria. Black garlic are rich in natural antioxidants, nutrients, and vitamins as compared to normal garlic and are produced by controlled fermentation of white garlic. Black garlic does not stink as white garlic but possesses enhanced bioactivity, making it more suitable for edible confectionaries. A patent (CN102763759A) was granted for a chewing gum made with black garlic, tea polyphenols, L-arabinose, sorbitol, soybean phospholipids, sodium erythorbate, xylitol, citric acid and the gum base according to weight ratio [136]. 

### 5.2. Breath-Freshening Agent/Toothpaste

Toothpaste is an oral hygiene product containing abrasive and other hydrocolloids suspended in a humectant matrix. The matrix may be added with therapeutic ingredients, surfactants, sweeteners and flavouring agents. Although garlic ingestion generates malodor owing to the loaded sulphur metabolites but fermented black garlic are non-odorous and do not generate bad breath might be a helpful alternative. A patent application (CN111888315A) for the toothpaste preparation method with BGE is under consideration. This invention claims anti-bacterial activity against many bacterial species responsible for dental caries and bad breath/halitosis [139]. Another patent was assigned for an antitoxic bactericidal toothpaste made out of GE for disinfecting the respiratory tract, oral cavity and claims to control bad breath [140].

### 5.3. Garlic Gel

Gel formulations are mucoadhesive forms of drug delivery system with advantages such as enhanced resident time, better absorption, higher accessibility and bioavailability of drug [141]. Powdered garlic was evaluated for compatibility with different gelling agents such as Carbopol and methyl cellulose and found effective in release of bioactive components even after 90 days and recommended for tongue ulcer healing. Menthol crystal enhances the better penetration and soothing effect, and Clove oil masks garlic’s odour and taste [142]. A worldwide patent (WO2009092387A2) was assigned for a formulation containing GE, gelling agent (polyacrylic acid and hydroxypropyl methyl cellulose) and carnation oil for use as mouth wash against clinical symptoms of gingivitis such as anti-acute and chronic inflammation and tooth pain relief [143]. Various innovative products made from the garlic are presented in Table 4.

## 6. Safety of Garlic 

Garlic’s value may lie in prevention rather than cure. The presence of volatile organosulfur compounds, including DATS, DADS, and DAS, is thought to be the primary cause of garlic’s health advantages and different garlic preparations contain different garlic constituents [44]. Garlic appears to be non-toxic when ingested in tiny amounts. However, consumption of a substantial amount of garlic, results in several adverse effects have been reported, including burning sensations, diarrhea and gastrointestinal difficulties. 

It has been suggested that the safety of garlic and its active components should be investigated when used in higher quantities. Daily and long-term consumption of natural products such as garlic extracts is essential for preventing oral pathologies. Hence, it is crucial to consider the safety aspects of the natural extracts. One of the significant difficulties involved with the long-term usage of any product is the general public’s attitude toward safety. Long-term supplement use increases toxicity concerns. The safety of all garlic preparations must be taken into account as part of the quality control process [92]. As previously stated, the ingredients of garlic preparations differ, necessitating toxicological testing of each product to verify its safety. Garlic products must be safe, stable and practical, according to makers. The examination of all products, including garlic, proposed for use in health promotion must include documentation of their safety and effectiveness. 

In another investigation, the safety aspects of enteric-coated products was also evaluated. The gastrointestinal mucosa was reddening after direct administration of pulverised enteric-coated goods. Oral administration of an enteric-coated tablet resulted in the epithelial cells degeneration at the top of crypts in the intestinal ileum. According to studies, while choosing a garlic preparation, consideration should be exercised in safety and effectiveness, as some preparations may have unfavorable consequences, such as gastrointestinal difficulties [116].

In current review some limitations are observed, firstly there are few clinical studies available which support the in vitro and in vivo study and most of the clinical trials reported in online database clinicaltrial.gov does not reported results of trial, therefore publications become the only way to review studies. Another limitation is that except for aged garlic extract, other garlic supplements have no toxicity or safety studies, and just a few, like an AGE, has clinical studies to back up their efficacy. Various toxicological investigations have proven the safety of AGE. Many toxicological and clinical trials of AGE, have found no negative effects [27]. A double-blind crossover study have demonstrated that AGE is safe: (1) acute and sub-acute toxicity tests chronic toxicity tests; (2) tests related to mutagenicity; (3) tests related to teratogenicity (segments I, II, and III); (4) toxicity test conducted by the U.S. Food and Drug Administration; (5) clinical trials investigated on 1000 subjects [147] and (6) tests related to general toxicity [42]. A human clinical trial (NCT03795636; Ahmad Elheeny, Minia University) reported, the efficacy of GE as an irrigant in pulpectomy of primary molars. 90 Children age ranged 4 to 6 years were selected with study period of 12 months, results of GE radiographic and clinical success rate were 72.7% and 80% for 3 months and 76.4%, 74.5% for 6 and 12 months, respectively. In comparison, NaOCl group shows 87.3% and 85.5% 3 months clinical and radiographic success rate, following 87.3% and 87.3% for 6 months and 89.1% and 87.3% for 12 months [148]. However, for future prospective more clinical trials, in vivo and in vitro studies must be conducted to discover potential bioactivities of garlic to alleviate oral pathologies. Based on the results of clinical studies, GE and AGE can provide a potent natural product with various preparations like gels, toothpaste mouthwash in treatment of various oral pathologies such as periodontitis and gingivitis. 

## 7. Conclusions

Garlic has remarkably high biological and medicinal properties since it is bestowed with a wide variety of bioactive compounds such as phenolics, essential oils, sulfur-containing compounds, flavonoids, volatiles, minerals and vitamins. In vitro and in vivo studies on antimicrobial activities of garlic against various pathogenic bacteria such as *Lactobacillus acidophilus* and *Streptococcus mutans*, anti-inflammatory, anti-cancerous and antioxidant activity against various oral pathologies periodontitis, dental caries, denture stomatitis. These studies were found to be supported by various human clinical studies reported garlic to be safe and effective in treatment of various oral pathologies. Garlic-based innovative products and allicin-based preparations such as chewing gums, toothpaste and gel was reported which can be possible sources of cost-efficient and consumer-friendly nutritive ingredients for ameliorating human oral health. However, there is an immense scope to utilize this property of garlic to treat several disease conditions. The results obtained in-vitro and intervention studies have been inconsistent in recognizing the distinct functional properties of each bioactive compound of GE and the strategy to enhance their bio-accessibility. There is relatively scanty information on the molecular mechanism of garlic bioactives like allicin. To understand the garlic extract action, its molecular mechanism must be investigated. Furthermore in-vivo and human clinical studies should be performed to consolidate the effect of garlic extracts on human health. Though there was no case of garlic extract toxicity, one recent report highlights the mucosal damage. Critical examination of all garlic-based products and their dosage is necessary for their safety and effectiveness. Based on these results, a policy must be formulated to use garlic in commercial products.

## Figures and Tables

**Table 1:** Bioactive constituents in garlic.

Variety	Type of Extract	Bioactive Compounds Identified	References
Raw garlic bulb	AGE & EGE	Sulphur containing compounds (2.3%) (Thiosulphinates such as allicin, allylmethyl-, methylallyl- and trans-1-propenyl-thiosulfinate); (OrganoSulphur volatiles such as DADS, DAS, DATS, sulfur dioxide, E/Z-ajoene, SAC, and S-allyl-cysteine sulfoxide (alliin)); (Vinyldithiins such as 2-vinyl-4H-1,3 dithiin)	[27,31]
Raw garlic bulb	AGE & EGE	Phenols (1.5%), (β-resorcylic acid, pyrogallol, gallic acid, rutin, protocatechuic acid and quercetin)	[27]
Raw garlic bulb	AGE & EGE	Saponins (diosgenin, gitogenin and β-cholorogenin)	[30]
Raw garlic bulb	AGE & EGE	Carbohydrate (starch, sucrose, glucose, fructose)	[27]
Raw garlic bulb	AGE & EGE	Fatty acids (palmitic acid, oleic acid, linoleic acid, linolenic acid)	[27]

**Table 2:** Biological activities of garlic in management of oral health.

Plant Source	Type of Extract	Bioactive Compounds Identified	Type of Study	Major Findings and Mechanism of Action	References
Antioxidant Activity					
Garlic	Lyophilized garlic powder	Allicin, ajoene, DAS, DADS, DATS, 2-VD	In vitro study on antioxidant activity of garlic and the mechanisms involved in this activity	Garlic samples depicted TPC (2.43–11.21), DPPH (0.05–0.58 mg GAE/100 g DW), ABTS (0.02–164.80 mg GAE/100 g DW) and FRAP (12.30–164.80 mM TEAC/10 mg DW). Allicin, ajoenes and 2-VD showed higher antioxidant activity compared to DAS, DADS and DATS.	[41]
Garlic	Lyophilized garlic powder	Allicin	In vitro study on antioxidant activities of garlic	TPC (1.48–3.48 mg GAE/g DW), DPPH (IC_50_: 6.25–33.28 mg/mL), ABTS (IC_50_: 11.46–46.53 mg/mL), Allicin content (127.33–165.43 mg/100 g DW)	[46]
Antimicrobial Activity					
Garlic	AGE EGE	Allicin and DAS	In vitro study of the efficacy of GE as antibacterial agents against periodontal pathogens including *Porphyromonas gingivalis* and *Aggregatibacter actinomycetemcomitans*	AGE with different concentrations—25, 50, and 75 μL showed 17 mm, 21 mm, and 26 mm zone of inhibition, respectively. The AGE showed higher bacteriostatic activity than EGE against the *P. gingivalis* with MIC determined at 16.7 μL/mL.	[52]
Garlic	Hydro alcoholic GE	Allicin and other thiosulfonates	Clinical study on efficiency of different concentrations (40 and 70%) of GE in alleviating of oral salivary bacteria in a culture medium of Trypticase Soy Agar (TSA) (Saliva samples: 40 Patients)	The 40% concentration at 30 s revealed a 78.5% reduction, and at 60 s, there was an 83.3% reduction in CFU. The 70% concentrations showed 86.6% reduction at 30 s and 90.9% reduction in CFU at 60 s.	[56]
Garlic	GE in ethanol, hexane, acetone, water diethyl ether	Organosulfur compounds alkaloids saponins flavonoids tannins steroids	Human clinical study on potential effect of GE for the treatment of biofilm-forming clinical pathogens *Lactobacillus acidophilus*, *Streptococcus sanguis*, *S. salivarius*, *S. mutans* and *Staphylococcus aureus* recovered from periodontal and dental caries	GE showed high antibacterial activity against *Staphylococcus aureus* and *Streptococcus mutans*. EGE were particularly more effective against the pathogens, probably due to the more phytochemical content in the EGE	[61]
Garlic	AGE	Allicin allicin thiosulfonates ajoene	In vitro study on evaluation of the antimicrobial activity of two garlic clones (1: purple and 2: white) crude extracts against nine oral Streptococci strains was carried out	The white garlic clone was more effective than the purple one. MIC ranged from 0.5–33.0 mg mL for purple clone and from 7.0 to 63.0 mg mL for the white clone. MBC ranged from 1.0 to 129.0 mg mL and from 9.0 to 129.0 mg mL regarding purple and white clones, respectively	[98]
Garlic	DMSO extracts of garlic bulbs with 1:1 Nano silver 25 nm	Allyl 2-propenethiosulfinate	In vitro study to evaluate the antibacterial property of nanosilver coupled edible plant extracts against *Streptococcus mutans*	The synergism of silver nanoparticles and GE on *S. mutans* isolates produced bigger-sized inhibition zones than GE alone and the MIC ranged between 52.5 ± 7.73 to 103.6 ± 5.91 mg/mL.	[99]
Antifungal Activity					
Garlic Green tea	AGE	Allicin	Randomized double-blinded controlled clinical trial aimed to evaluate and compare the antimicrobial efficacy of green tea, garlic with lime and 0.05% NAF mouth rinses against *Candida albicans* (45 Patients, Time period: Once daily for 2 weeks)	Colony count of *C. albicans* NAF Mean baseline, 7.7 × 10^4^ CFU/mL Mean postrinse, 4.5 × 10^4^ CFU/mL Garlic with lime Mean base line, 7.1 × 10^4^ CFU/mL Mean postrinse, 4.3 × 10^4^ CFU/mL Green tea Mean base line, 6.4 × 10^4^ CFU/mL Mean Post-rinse, 2.3 × 10^4^ CFU/ml	[68]
Garlic	Paste		Human clinical study (randomized trial) aimed to evaluate the clinical efficacy of topical garlic paste in comparison with clotrimazole solution in 56 patients for 14 days with oral candidiasis	Percentage of patients with clinical response: The percentage of patients cured was 50%, improved was 36.7% whereas, 13.3% showed no change in clotrimazole group. For garlic group, the percentage of patients cured was 61.5%, improved was 38.5%, every patient showed positive improvements by garlic paste.	[69]
Garlic	AGE	Allicin	Randomized clinical trial aimed to compare the antifungal effect of nystatin (N) mouth wash with AGE on denture stomatitis (4 Weeks, 40 Patients)	Mean width of erythema At the start of treatment, ✓ N:3.03 ± 1.03 cm ✓ GE: 3.63 ± 1.21 cm After treatment, ✓ N: 0.08 ± 0.18 cm ✓ GE: 1.09 ± 0.5 cm Mean length of erythema At the start of treatment, ✓ N: 3.61 ± 0.88 cm ✓ GE: 3.53 ± 1.116 cm After treatment, ✓ N: 0.11 ± 0.21 cm ✓ GE: 0.99 ± 0.34 cm	[70]
Garlic	AGE	Allicin	In vitro study aimed to evaluate the efficacy of garlic and propolis extracts against *Candida albicans* and compare it with Amphotericin-B as control at 3 different concentrations.	Anticandidal actions at different concentration at 24 and 48 h duration (i) Amphotericin-B (Control) 24 h 48 h 10% 0.002000 0.000667 (ii) Propolis 24 h 48 h 10% 0.056000 0.098667 20% 0.082333 0.133333 30% 0.003333 0.131000 (iii) Garlic 24 h 48 h 10% 0.000667 0.004000 20% 0.000000 0.000000 30% 0.000000 0.000000	[72]
Garlic	Essential’s oil	DAS Methyl allyl disulfide Diemethyl trisulfide	In vitro study aimed to evaluate the effect of garlic essential oil against *Candida* species	The MIC was lowest for *C. albicans* (0.4 µg/mL) followed by *C. tropicalis* (0.5 µg/mL) and *C.glabrata* (0.6 µg/mL). Similarly, MFC was lowest for *C. albicans* (0.7 µg/mL) and similar for *C. tropicalis* (0.8 µg/mL) and *C. glabrata* (0.8 µg/mL). *C. albicans* has more susceptibility to garlic essential oil.	[74]
Garlic	Powder	Allicin Lycopene Monohydrate Selenium Dioxide Vitamin A Vitamin C Vitamin E Zinc Sulphate	Clinical study aimed to assess the effect of heat killed *Lactobacillus acidophilus* as probiotic and GE as a prebiotic on salivary *Candida albicans*	*Candida albicans* counts: Control: 42 GE + LB: 3.33–1.8 *C. albicans* bioform forming ability Control: 5.0 GE + LB: 0.31–0.26	[100]
Antiviral Activity					
Garlic	NS	allicin	In vitro rabbit skin cells and plaque count assay	Dose of 0.015 mg/mL and higher significantly reduce the HSV-1 population	[81]
Garlic	Fresh GE (juice), polar fraction and garlic associated compound	In GE: Thiosulfinates (Allicin) ailyl and aliyi methyl thiosulfinates and trans-1-propenyl allyl thiosulfinate In aqueous (polar) fraction: y-giutamyl-S-trans-1-propenyl-cysteine, y-glutamyl-SAC and y-gluta- myi-S-methylcysteine	In vitro plaque assay techniques on HeLa and Vero cells Cytotoxicity assay	The compounds were toxic against HSV-1 & HSV-2. Highest viricidal activity was in ajoene followed by allicin, allyl methyl thiosulfinate and methyl allyl thiosulfinate	[82]
Garlic	Oil using hydro-distillation	3,3’-thiobis-1-Propene, Disulfide, 3 Methyl-trans-propenyl-disulfide, cis-Propenyl methyl disulphide, Propanedioic acid, Dimethyl trisulfide, Limonene, 8 Di-2-propenyl disulphide, Methyl-2-propenyl trisulfide, 3-vinyl-[4H]-1,2-dithiin, 2,4,5,6-Tetramethylpyrimidine, 2-vinyl-[4H]-1,3-dithiin, Di-2-propenyl trisulfide, Isobutyl isothiocyanate, 3,3’-thiobis-1-propene, 2,3-Dicarboxythiophene, Diallyl tetrasulphide, Diallyl pentasulfide, Diallyl hexasulfide, Methyl allyl, Mentasulfide, Methyl allyl hexasulfide	In vitro study Vero cells in cytopathicity assay antiviral activity against HSV-1 under	Increased the longevity of cells treated with GEO (Garlic essential oil)	[83]
Garlic	AGE and alcoholic extract	-	In vitro Hela cell culture	Very effective against HSV-1 Required higher dose for cytotoxic effect compared to antiviral effect	[84]
Garlic	NS	NS	In-vitro cells plaque reduction and early antigen assay	Antiviral activity against CMV was in dose dependent manner and author recommended as prophylactic use	[101]
Anti-Inflammatory Activity					
Garlic	AGE tablets (300 mg AGE powder)	SAC, S1PC and SAMC	Randomized controlled double-blind clinical study aimed to assess the long-term efficacy of AGE for the treatment of periodontitis (201 participants, 18-month study period)	Prevent or improve periodontal disease. SAC and SAMC inhibit LPS-induced inflammation in human gingival epithelial cells by suppressing intercellular adhesion molecule-1 expression and IL-6 secretion.	[90]
Garlic	AGE tablets	-	Randomized controlled clinical study to assess the effectivity of AGE extract in reducing gingivitis and gingival bleeding (151 participants, 4-month study period)	Decrease in Gingival bleeding index and Gingival index score with treatment of AGE is observed compared to placebo group (*p* < 0.001).	[91]
Garlic	Aged garlic extract (AGE > 10 months in ethanol)	SAC, S1PC and SAMC	In vitro study aimed to examine the role of SAC, S1PC and SAMC in AGE induced anti-inflammatory effects	Alleviation of gingivitis by reducing the inflammation markers IL-1β, IL-2, IL-6, IFN-γ	[102]
Anticancer activity					
Garlic	SAC standard purchased from LKT laboratories (USA)	SAC	In vivo study on mouse xenograft model	SAC showed anti-oral cancer activity by suppressing the osteopontin and N-methylpurine DNA glycosylase and by inhibiting the APK/ERK and phosphatidylinositol-3-kinase/Akt signalling pathways	[99]
Garlic	Allicin standard extracts	Allicin	In vitro primary oral tumors from oral squamous cell carcinoma patients were collected	Allicin alleviates the pain in oral squamous cell carcinoma by attenuating the TNF-α, pain mediators, endothelin and IL-8	[103]

**Table 3:** Role of GE and its bioactive to alleviate oral pathologies.

Plant Source	Type of Extract	Bioactive Compounds Identified	Disease Studied	Major Findings and Mechanism of Action	References
2.5% garlic, Karnataka, India	100 mL AGE	Allicin and thiosulfinates	Tooth decay	Growth of cariogenic bacteria, *Streptococcus* *mutans* were inhibited at MIC range of 4–32 mg/mL	[117]
Chinese garlic cloves	100 g diethyl ether	Allicin and DAS	Periodontal	*Porphyromonas gingivalis*, an anaerobic, gram-negative pathogen linked to chronic periodontitis, has the lowest allicin sensitivity (2, 400 μg/mL).	[83,118]
Garlic, Manila	70% aqueous	Allicin	Pulpitis	GE 70% was able to disrupt and prevent the production of *Enterococcus faecalis* biofilm in root canals.	[119,120]
Garlic, Changsha, China	Garlic plaster including 0.1% garlicin	Garlicin	Recurrent oral ulcers	Overall effective rate was 100%, while the complete effective rate was 83.3%.	[121,122]
Garlic, Chenzhou, China	Allicin adhesive tablets contained 5 mg allicin magnesium stearate, sodium carboxymethylcellulose, and flavoring additives	Allicin	Recurrent aphthous ulceration	Allicin adhesive tablets reduced ulcer size (72.5%) and pain (75.7%) dramatically.	[116]
Fresh garlic, Provo, USA	Aqueous alcohol	Diallyl thio-sulfinate (allicin), allyl methyl thiosulfinate, methyl allyl thiosulfinate, ajoene, alliin, deoxyalliin, DADS, and DATS	Herpes simplex virus type 1,2, Parainfluenza virus type 3, vaccinia virus, vesicular stomatitis virus type 2	Disruption of the viral envelope and cell membrane to prevent virus entry	[54,79]
Garlic, Davangere, Karnataka, India	Garlic pearl and 0.25% garlic oil	Dimethyl trisulfide	Oral submucous fibrosis	95.68% reduction in burning sensation and a 5.37 mm increase in mouth opening	[123]
Garlic, Mangalore, Karnataka, India	Garlic paste with one drop of 2% lignocaine jelly and Clotrimazole solution 1% *w*/*v* (2–4 drops),	Ajoenes, allyl sulfides	Oral candiasis	More than 50% reductions in the colony count, 61.5% complete eradication of lesions	[72]

**Table 4:** Various innovative products developed from garlic.

Type of Product	Product	Patents	Plant Part Used	Intended Use	Reference
Toothpaste	Toothpaste with BGE	CN111888315A	Black garlic blub	To realise the broad-spectrum sterilization effect targeting various oral pathogens.	[139]
	Garlic antitoxic bactericidal toothpaste	CN1555777A	Garlicin and Garlic oil	To disinfect respiratory tract and oral cavity and treating foul breath	[140]
Garlic gel	Garlic gel-Pharmaceutical composition containing a GE	WO2009092387A2	AGE	Anti-Acute and Chronic inflammation and ToothPain Relief	[143]
Chewing Gum	Black garlic chewing gum	CN102763759A	Black garlic blub	To promote elimination of human radicals, enhance human immunity, stabilize blood pressure, lower blood lipid and blood sugar, lower cholesterol and reduce weight, and fine health caring effect	[144]
Mouth fresheners	oral cavity nursing agent	CN103384526B	Garlic clove	To improve the composition of oral health and prevent dental caries, or calculus dentalis, treating oral malodour and halitosis	[145]
Pharmabiotic strips	Adherent oral pharmabiotic delivery strip	US 20200155447 A1	AGE	An oral pharmabiotic system is disclosed for improving oral, dental, and systemic health by repopulating and reshaping the flora within a patient’s oral environment in a manner that overcomes the deficiencies of prior oral probiotic products.	[146]
